# Towards Quantification of Inflammation in Atherosclerotic Plaque in the Clinic – Characterization and Optimization of Fluorine-19 MRI in Mice at 3 T

**DOI:** 10.1038/s41598-019-53905-9

**Published:** 2019-11-25

**Authors:** Emeline Darçot, Roberto Colotti, Maxime Pellegrin, Anne Wilson, Stefanie Siegert, Karima Bouzourene, Jérôme Yerly, Lucia Mazzolai, Matthias Stuber, Ruud B. van Heeswijk

**Affiliations:** 1 Department of Radiology, Lausanne University Hospital (CHUV) and University of Lausanne (UNIL), Lausanne, Switzerland; 2 Division of Angiology, Heart and Vessel Department, Lausanne University Hospital (CHUV), Lausanne, Switzerland; 3 Flow Cytometry Facility, Department of Formation and Research, University of Lausanne (UNIL), Epalinges, Switzerland; 4 Center for Biomedical Imaging (CIBM), Lausanne and Geneva, Switzerland

**Keywords:** Diagnostic markers, Diagnostic markers, Atherosclerosis, Magnetic resonance imaging, Translational research

## Abstract

Fluorine-19 (^19^F) magnetic resonance imaging (MRI) of injected perfluorocarbons (PFCs) can be used for the quantification and monitoring of inflammation in diseases such as atherosclerosis. To advance the translation of this technique to the clinical setting, we aimed to 1) demonstrate the feasibility of quantitative ^19^F MRI in small inflammation foci on a clinical scanner, and 2) to characterize the PFC-incorporating leukocyte populations and plaques. To this end, thirteen atherosclerotic apolipoprotein-E-knockout mice received 2 × 200 µL PFC, and were scanned on a 3 T clinical MR system. ^19^F MR signal was detected in the aortic arch and its branches in all mice, with a signal-to-noise ratio of 11.1 (interquartile range IQR = 9.5–13.1) and a PFC concentration of 1.15 mM (IQR = 0.79–1.28). Imaging flow cytometry was used on another ten animals and indicated that PFC-labeled leukocytes in the aortic arch and it branches were mainly dendritic cells, macrophages and neutrophils (ratio 9:1:1). Finally, immunohistochemistry analysis confirmed the presence of those cells in the plaques. We thus successfully used ^19^F MRI for the noninvasive quantification of PFC in atherosclerotic plaque in mice on a clinical scanner, demonstrating the feasibility of detecting very small inflammation foci at 3 T, and advancing the translation of ^19^F MRI to the human setting.

## Introduction

Atherosclerosis manifests as a chronic inflammatory process that affects the inner layer of arteries and that leads to the formation of plaque. Several medical imaging modalities can be used to assess atherosclerotic plaques^1,2^. Most techniques that are used in clinical routine only inform on the anatomical features induced by the disease (such as vessel wall thickness, degree of stenosis, necrotic core, calcifications and fibrous cap thickness), which appear at the later stages of plaque formation. At the early stages, atherosclerotic plaques grow and evolve below the clinical detection threshold. Given that inflammation plays a crucial role in plaque formation throughout the whole course of the disease^3^, the degree of inflammation in plaque might therefore be the ideal target for a better and earlier monitoring of atherosclerosis. Several techniques have previously been described for the detection of inflammation^4–7^, but these may involve ionizing radiation, contrast agents that clear relatively rapidly from tissues, or stay deposited in the tissue after the inflammation clears; they might therefore pose significant radiation or quantification challenges when applied for monitoring atherosclerosis over time.

Recently, fluorine-19 (^19^F) magnetic resonance imaging (MRI) of perfluorocarbon (PFC) emulsions has been increasingly used for cell tracking and inflammation imaging due to its high specificity and the biological and chemical inertness of PFCs^8–10^. PFCs are highly suitable biomarkers for inflammation: since they are phagocytized by immune cells after intravenous injection^11^, they enable the specific imaging of the carrier immune cells with a high contrast-to-noise ratio (CNR) since there is negligible endogenous ^19^F MR signal due to the low natural abundance of ^19^F atoms in the body. Furthermore, PFCs are interesting candidate biomarkers for studies that require longitudinal monitoring, given that they can only be carried by living cells, and clear out of a tissue once released by their phagocytosing immune cell^12^. ^19^F MRI of PFCs has therefore already been used to image and monitor inflammation in a wide range of animal disease models such as cardiac and cerebral ischemia^9^, myocarditis^13^, pneumonia^14^, multiple sclerosis^15^, and atherosclerosis^16^, mostly on preclinical high-field MRI scanners. Independently, several PFCs have furthermore already been used in clinical trials as blood volume expanders^17^, while the PFC perfluoropolyether (PFPE) was recently used in a phase I clinical trial for stem cell monitoring during cancer treatment^18^.

However, the translation of ^19^F MRI for inflammation monitoring into clinical practice still requires a large research effort in order to overcome its two main hurdles: regulatory approval of the PFCs and the loss of ^19^F MR sensitivity at the lower magnetic field strength of clinical scanners.

To address this second hurdle, the quantification and monitoring of inflammation in an animal model on a clinical scanner is the logical next step toward the ultimate goal of using ^19^F MRI as a noninvasive, highly specific, and quantitative clinical tool for tracking and monitoring of inflammation. To compensate for the loss of sensitivity due to the translation from ultrahigh-magnetic-field animal scanners to lower-magnetic-field clinical scanners, both the MRI acquisition and reconstruction should be optimized. This was already investigated in pigs and mice for myocardial infarction^19,20^ without quantification of the ^19^F concentration at the inflammation site, and while it was quantitatively explored in a pair of mice for cancer^21^ and *ex vivo* for cell tracking^22^, it remains to be studied for small low-signal inflammation foci such as atherosclerotic plaques.

In the case of atherosclerosis, the physiological mechanisms behind the detected ^19^F signal are currently unclear: knowledge of the specific immune cell populations that take up the PFCs and their distribution at the plaque site would help improve our understanding of the recruitment of immune cells in atherosclerosis, and may allow us to increase the precision and reproducibility of the ^19^F MR signal measurement. A well-suited technique for quantifying these immune cell populations is imaging flow cytometry (IFC)^23^, which benefits from the combination of the sample sizes and acquisition rates of conventional flow cytometry as well as the specificity and spatial resolution of microscopy.

The goals of this study were thus two-fold: 1) to demonstrate the feasibility of quantitative ^19^F MRI in very small inflammation foci on a 3 T clinical scanner, and 2) to characterize and quantify immune cells that incorporate PFCs. To this end, we optimized the ^19^F MRI acquisition for imaging of atherosclerotic plaques in a murine model of atherosclerosis. The acquisition, reconstruction and analysis of the *in vivo* datasets were performed in parallel of the IFC. After the *ex vivo* acquisitions, the immune cell populations identified by IFC were then targeted to perform the immunohistochemistry analysis.

## Results

### *In vivo* imaging of inflammation

**Figure 1:**
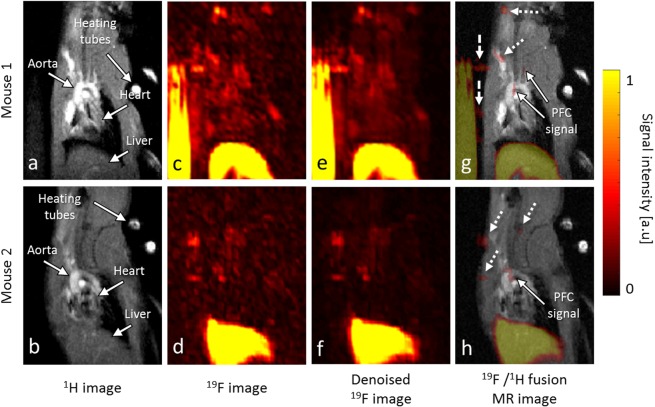
*In vivo* MRI of PFCs in mice with atherosclerotic plaque at 3 T. (**a**,**b**) *In vivo* double oblique ^1^H MR images through the liver, the heart and the aorta. (**c**,**d**) The corresponding standard ^19^F MR images, and (**e**,**f**) the corresponding denoised ^19^F MR images. Both sets of images illustrate the patchy distribution of the PFC at the aorta and its branches. (**g**,**h**) The ^19^F denoised/^1^H MR fusion image shows that the small PFC signals are mainly located in the aortic arch and the brachiocephalic, left carotid and left subclavian arteries (solid arrows). ^19^F signal was also observed in the subcutaneous fat, where the anesthetic isoflurane accumulates (dotted arrows). In the top row, the high PFC concentration and motion (due to the breathing of the mouse) of the reference tube caused several ^19^F motion artifacts next to it (dashed arrows).

^19^F MR images showed a patchy signal distribution at the level of the aortic arch and its branches (Figure 1). In addition, a strong ^19^F signal was observed in the liver (Figure 1c,d) as well as a low-intensity ^19^F signals in the subcutaneous fat and spinal cord (Figure 1e,f), which were assigned to the anesthetic isoflurane^24^ and the reticuloendothelial system. In images reconstructed with standard Fourier transform, the average SNR of the ^19^F signals identified as atherosclerotic plaques was 11.1 (interquartile range IQR = 9.5–13.1)and 47.9 ± 23.2 in the liver (range 21.2–107.3). The MRI-derived PFC concentration measurement in the plaques was 1.15 mM (IQR = 0.79–1.28), while the plaque ^19^F signal integral (i.e. SNR × volume) was 37.8 ± 22.9 mm^3^ (range 4.3–74.7 mm^3^). Motion artifacts from the high PFC concentration in the reference tube were always present in the 3D ^19^F MR images, but due to the placement of the tube diagonally above the mouse, they never projected into the thorax. An SNR of 3.0 ± 0.9 (range 1.2–4.6) was measured in the aortic arch regions without specific ^19^F signal, which was significantly different from the plaque SNR (p < 0.001).

**Figure 2:**
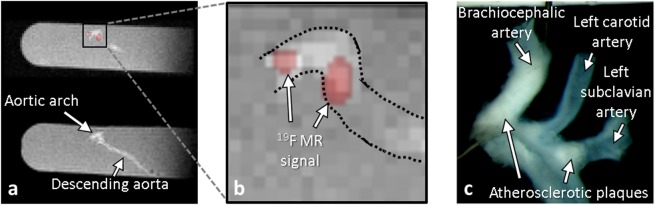
*Ex vivo* confirmation of PFCs infiltration in suspected atherosclerotic plaques. (**a**) Multi-planar reformatted ^1^H (bottom) and fused ^19^F/^1^H (top) images of the entire excised aorta of an ApoE^−/−^ mouse. (**b**) The zoomed inset shows ^19^F patches in the aortic arch (dotted lines are the outline of the aorta on the ^1^H image). (**c**) En-face photo of the aortic arch, showing atherosclerotic plaque as white patches that visually match the location of the ^19^F MR signals observed in b (white arrows).

In the denoised images, the ^19^F signals were visually more conspicuous: they stood out more against the background noise and isoflurane signals compared to the standard images. However, despite the light regularization weights, no ^19^F signal was observed in the aorta and its branches in four out of the thirteen mice in the denoised images. These four animals were excluded from the subsequent MRI-derived PFC concentration and plaque volume comparisons between the denoised and standard images. The plaques in the denoised images resulted in an MRI-derived PFC concentration measurement that was 11% lower than that of the standard images (1.02 mM (IQR = 0.84–1.35) vs. 1.18 mM (IQR = 1.02–1.40), respectively, p = 0.01). The plaque volume segmented in the denoised images was also 30% smaller than in the standard images (2.58 ± 0.94 mm^3^ vs. 3.67 ± 1.23 mm^3^, i.e 4.2 ± 1.5 pixels vs. 6.0 ± 2.0 pixels, respectively, p = 0.003).

### *Ex vivo* confirmation

3D *ex vivo* MRI confirmed the presence of atherosclerotic plaques within the aorta (Figure 2a,b). ^19^F signal was detected at the locations where the MR *in vivo* images with standard reconstruction showed the presence of atherosclerotic plaques in the aortic arch and its branches. The plaque surface estimated from the en face photographs was 0.5 ± 0.3 mm^2^ (Figure 2c). The measured ^19^F MR plaque volume did not correlate with the photographed plaque surface (p > 0.1 for both MR reconstruction techniques).

### Immune cell populations

**Figure 3:**
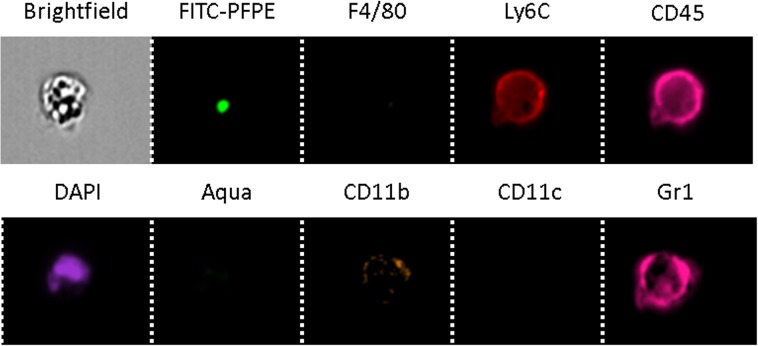
Example of the imaging flow cytometry channels of a single cell. The bright-field image of this neutrophil provides information about cell size and smoothness of the cell surface. In this case, FITC-PFPE aggregated in a single small compartment of the cell. DAPI is a nuclear stain, while Aqua reacts with free amines both in the cell interior and on the cell surface and is used to mark dead cells. When present, the other antibody conjugates were attached to the cell surface, as shown by the bright signal at the edge of the cell on these images.

**Figure 4:**
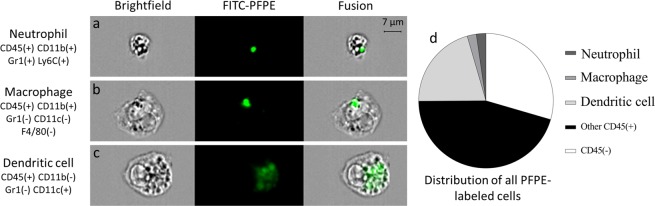
Microscopic visualization and characterization of individual immune cells that took up the PFCs. For clarity, only one anatomical and one fluorescent channel are shown in this image - from left to right: brightfield (gray), FITC-PFPE (green) and their overlay. Examples of (**a**) a neutrophil, (**b**) a macrophage and (**c**) a dendritic cell with internalized PFCs. While the PFC signal appeared to aggregate in one small 2–3 μm compartment in neutrophils and macrophages, the uptake seemed more diluted and scattered in the dendritic cells. (**d**) Dendritic cells made up a high percentage of the FITC-PFPE labeled cells (20.6%), while a similarly low percentage of macrophages and neutrophils was measured (∼2.3%). Almost half of the CD45 positive events (45.4%) were unspecified leukocytes, while 29.5% of the FITC-PFPE labeled cells were CD45-, i.e. not leukocytes.

IFC enabled the visualization and characterization of the different immune cell populations that took up the PFCs based on the antibodies expression (Figure 3). Among the analyzed living cells, 770 cells were FITC-PFPE+ (1.4% of the counted cells) and 538 leukocytes were FITC-PFPE+ (1.3% of the detected leukocytes). The identified immune cells among the FITC-PFPE+ cell population were mainly neutrophils, macrophages and dendritic cells at a ratio of 1:1:9 (Figure 4). No PFC was detected in any dead cells (Aqua+). Small PFC spots were observed in neutrophils (area = 3.7 ± 1.8 µm^2^, relative brightness = 16.5 ± 9.8, Figure 4a) and in macrophages (area = 4.6 ± 4.2 µm^2^, relative brightness = 16.4 ± 13.7, Figure 4b) as opposed to the more diffuse signal pattern that characterized the dendritic cells (area = 22.0 ± 18.0 µm^2^, relative brightness = 70.0 ± 54.9, Figure 4c). The total PFC signal per cell (area × relative brightness) was ∼20 × higher for dendritic cells than for macrophages and neutrophils. The majority of the PFCs (97%) were observed inside the cells, as opposed to inside spherical structures attached to the outside of the cells. The control study showed that non-fluorescent PFC-labeled cells expressed significantly smaller signals at the FITC wavelength than the cells labeled with FITC-PFPE. This indicated that the autofluorescence signal originating from the PFCs and the background was weak compared to the fluorescent signal of the FITC label, which confirms the specificity of the IFC modality.

### Plaque composition

**Figure 5:**
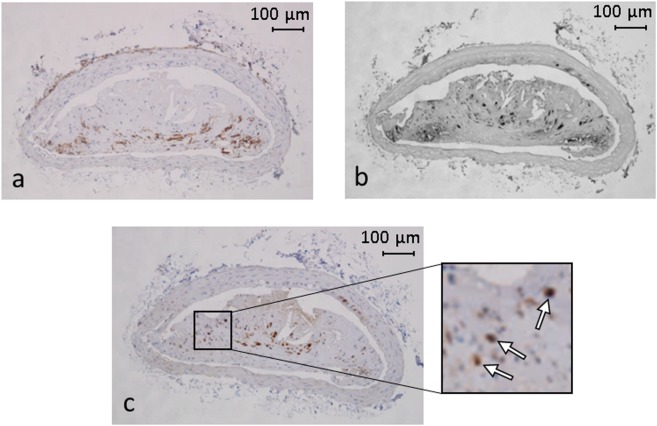
Representative images of cross-sections of brachiocephalic artery immunostained for leukocytes, macrophages and dendritic cells. (**a**) CD45, (**b**) Mac-2 and (**c**) S100 staining that revealed leukocytes (brown stain), macrophages (black stain) and dendritic cells (brown stain), respectively. Leukocytes and macrophages were quantified by determining the percentage of positive stained area of the total plaque area while dendritic cells were individually counted (white arrows in inset). No signal was detected when slices were stained with appropriate isotype controls (data not shown).

Immunostaining provided a detailed overview of the composition of the plaques from ApoE^−/−^ mice, which exhibited a high leukocytes, macrophages, and dendritic cells content (Figure 5). Quantitative analysis of leukocyte (CD45) and macrophage (Mac-2) immunostainings revealed that the percentage of CD45- and Mac-2-positive areas per plaque were 12.2% (IQR = 8.3–14.6) and 19.6 ± 5.4%, respectively, while the number of S100-positive dendritic cells per plaque was 514 ± 244 mm^−2^. The total plaque area, measured for each immunostaining analysis was 0.13 ± 0.07 mm^2^, 0.16 ± 0.08 mm^2^ and 0.13 ± 0.06 mm^2^ for the leukocyte, macrophage and dendritic cell analyses, respectively. No signal was detected when slices were stained with appropriate isotype controls (data not shown). The plaque surface from the en face photos (Figure 2c) correlated significantly with the measured plaque area from those immunostaining analysis (r > 0.67, p < 0.02). ^19^F MRI metrics did not significantly correlate with any of the histological metrics (all p > 0.05).

## Discussion

We successfully used ^19^F MRI *in vivo* to detect PFC in atherosclerotic plaques in a murine model on a 3 T clinical scanner. The immune cell populations and the plaques that internalized the PFCs were characterized at the cellular level with IFC and immunohistochemistry.

The *in vivo* ^19^F MRI showed a patchy PFC distribution in the aortic arch and its branches, particularly at the locations where the endothelial cells are known to experience turbulent flow and the likelihood of atheroma formation is higher^25^, such as in the inner curve of the aortic arch and slightly above the root of the brachiocephalic, left subclavian and left carotid arteries, as commonly observed in mouse atherosclerosis studies^16,26^. Given that the sensitivity of ^19^F MRI for the detection of atherosclerotic plaque was investigated with a control group in a previous study, and no ^19^F MR signal was found near the great vessels in this group^16^, no control group was included in this study and therefore no receiver operating characteristic curve (ROC) analysis was performed. Morover, mice without atherosclerotic plaque do not accumulate any ^19^F signal in their aortas^16^, and ^19^F signal was detected in the major vessels of all mice with atherosclerosis in this study. Furthermore, the various signals not originating from the injected PFC were consistently limited to the regions of the reference tube (motion artifacts from its strong signal) and the subcutaneous fat (isoflurane), and never projected onto the vasculature of interest. Moreover, the previous study^16^ demonstrated with immunofluorescent histology that PFC is indeed taken up by the leukocytes. It is therefore very likely that ^19^F MRI can be used to detect atherosclerotic plaque in mice at 3 T with very high sensitivity and specificity, although when designing new experiments, the possibility of these confounding signals and artifacts should be taken into account.

A strong ^19^F signal was observed in the liver, where Kuppfer cells take up PFCs^27,28^. As reported in other studies^13,24,29^, low-intensity ^19^F signal originating from the anesthetic isoflurane was observed in the subcutaneous fat. While isoflurane signals have to our best knowledge never been reported at the level of heart and great vessels, they do decrease the specificity of ^19^F MRI, although the confounding visualization effect was reduced by thresholding the ^19^F data. This is also supported by the measured SNR in the aortic arch next to the plaques, which was below the Rose criterion of SNR = 4, meaning that it cannot be distinguished from noise^30^. Moreover, when isoflurane is in- and exhaled through the lungs, it is in a gas phase and its apparent relaxation times are too short to be detected with a TSE pulse sequence^31^, which means that there will be no confounding signal in the lungs. Isoflurane was used in this study because of the need for relatively long anesthesia and the unfeasibility of re-injection during scans, but future studies that aim to maximize specificity and have a limited total anesthesia duration should consider an injected anesthetic such as ketamine-xylazine. ^19^F signal was also observed in the vertebral column, which is consistent with recent demonstrations of PFC accumulations in the vertebral bone marrow^15^.

The denoised ^19^F images resulted in both lower measured concentration and plaque volume than the standard images. Since a low regularization weight that results in very little smoothing was used for the denoising, regularization smoothing is unlikely to be the main cause of this decrease. Interestingly, the relative decrease in plaque volume was almost three times larger than that of the concentration. This suggests that the signal removed by the denoising filter was low-intensity motion blur with an intensity just above the noise threshold, since the ^19^F MR acquisitions were performed without ECG triggering. Two of the four mice in which no ^19^F signal was found in the aortic region in the denoised images had the lowest SNR in both the liver (less than half the average) and the plaques in the standard images, indicating that these mice might have received or taken up lower doses of PFC. The other two mice might have had less immunologically active plaques, since they respectively had very low plaque SNR and the lowest plaque/liver SNR ratio in the regular images. While the denoising technique resulted in four false negative results, it might improve avoiding false positive results, although this was not demonstrated in this study. Therefore, while the denoising technique improved the conspicuity of the plaques, at this stage it has to be considered as an additional source of information on the segmented plaque size and MRI-derived PFC concentration rather than a replacement of the standard reconstruction technique. However, given that it is a post-processing technique, it does not interfere with the acquisition protocol.

Both the *ex vivo* MRI findings and the en face photos visually confirmed the presence of the atherosclerotic plaques in the aortic arch and its branches that were observed and characterized with the *in vivo* experiments. We did not perform quantitative measurement (SNR, signal integral, etc.) on the ex-vivo ^19^F MR images for several reasons. In the past we have observed in several studies when scanning very small objects and structures floating in a watery solution in a clinical scanner that the images are much noisier and more blurred than we would expect, as can also be seen in the ex-vivo MR images in this study. We attributed this to the watery solution surrounding the samples, which does not prevent gradient vibrations from moving the sample and may permit standing waves in the water that cause artifacts. This is indirectly confirmed by the fact that when we embed very small samples in agar gels, the noise goes down and images are sharper. This gel embedding could not have been used in this study because of the need of fixation and subsequent histology. There was furthermore a relatively long time delay of 16 weeks between the fixation and *ex-vivo* scanning of most of the samples, since we waited for all batches of Group A to have been euthanized before starting the *ex-vivo* analysis (Group A consisted of three batches that arrived and thus started their diet at different time points). During this time the PFC content might have decreased somewhat, although the stability of PFC in formaldehyde-fixated samples has not been documented. Finally, since fixation changes the relaxation times of the PFC, the scan parameters would need to be slightly adapted to be optimal.

The IFC analysis was successfully used to identify and quantify the immune cell populations that internalized the FITC-PFPE nanoparticles in the vessel wall of the aortic arch and its branches. As already observed by van Heeswijk *et al*.^16^, the FITC-PFPE uptake appeared to be unspecifically phagocytic. Given the highly specific cellular imaging component of IFC, an accurate analysis was still possible despite the low FITC-PFPE+ cell count. The atherosclerotic plaques analyzed in this study contained significantly more dendritic cells than macrophages and neutrophils, suggesting that they are advanced plaques, as described in previous studies^32^. Given that dendritic cells also took up over 20 times more FITC-PFPE per cell than macrophages and neutrophils, it appears that in this mouse model of atherosclerosis, the ^19^F signal was almost exclusively generated by dendritic cells. However, this might also partially depend on the strategy that was used for the population identification with IFC: our macrophage gating was relatively restrictive since for example the population that expresses F4/80 (mostly tissue resident macrophages) was excluded. We did not observe any FITC-PFPE+ dead cells. As also seen with NMR spectroscopy by Rose *et al*.^33^, this further supports the hypothesis that the death of PFC-loaded cells causes the rapid dispersion and exhalation of the engulfed hydrophobic nanoparticles^18^. Surprisingly, a substantial amount of CD45- cells were FITC-PFPE+. Since CD45 is a general leukocyte marker, these cells might for example be leukocytes that lost their receptors due to the digestion process, or fibroblasts, a type of stromal cells whose role in expressing immune response in the inflamed heart has been demonstrated by Kania *et al*.^34^ The FITC-PFPE+ compartment that was found attached to the outside of a small percentage of cells might be debris from other cells sticking to the cell membrane. We observed a diffuse FITC-PFPE signal pattern in dendritic cells, as opposed to the smaller FITC-PFPE spots in macrophages and neutrophils, indicating that they might use different compartmentalization mechanisms. Finally, although it has already been observed that there are limited numbers of aortic leukocytes that can be isolated for cell sorting analysis^35^, the low amount of FITC-PFPE+ cells might be further explained by the enzyme digestion mechanisms, which may affect the expression of the FITC-PFPE antigen, as well as by the possible faster clearance of the FITC fluorophore than the rest of the nanoparticle^36^.

The immunohistochemistry analysis confirmed the IFC findings of the significant presence of macrophages and dendritic cells in the plaques. Nevertheless, given that the selected antibodies used to define the cells have different expression frequencies, binding strengths, and quantification methods, the populations cannot be directly compared, and the IFC finding that dendritic cells outnumbered the macrophages cannot be confirmed.

No linear correlation was found between ^19^F MRI-derived metrics (PFC concentration, ^19^F SNR, ^19^F signal integral) and any of the histological metrics (plaque size and immune cells plaque content) or the en face photographs. This might have several causes, such as the relatively low ^19^F SNR and animal numbers, as well as the limited portion of the plaque that was analyzed with immunohistochemistry: only three 3 µm thick slices were analyzed per plaque, which might not be sufficient to provide a reliable depiction of the composition of the whole plaque. The absence of correlation in plaque volume between the different modalities and ^19^F MRI-derived metrics might also be explained by the small number of pixels that were measured. Interestingly, the en face photo plaque surface correlated with the measured plaque size (i.e. area) in immunohistochemistry. No correlations were performed between ^19^F MRI-derived metrics and IFC results given that IFC provided a global, and not individual, quantification of the immune cell populations over all aortas.

The absence of validation of the quantification of the ^19^F MRI signal by a reference technique such as histology (i.e. a strong correlation between the two metrics) leaves the accuracy of the inflammation quantification at this size and magnetic field strength by ^19^F MRI an open question. However, it is well established that ^19^F MRI provides an accurate linear fit for a wide range of concentrations and SNR measurements in both phantom studies and disease models^9,37–39^. Since there is only a mild influence of the change in relaxation times between tissues on the SNR quantification^38^, ^19^F MRI allows for a direct quantification of the PFC concentrations and volumes, assuming that the PFC concentration is directly linked to the leukocyte concentration. While the amount of PFC that a specific cell type takes up varies up to an order of magnitude, as evidenced by the standard deviation being nearly as large as the cellular uptake itself when measured with IFC, this should still allow a reasonably accurate estimation of the immune cell population.

The main limitation of this study is the low concentration of PFC in chronic inflammation combined with the relatively low sensitivity of ^19^F MRI. This results in the abovementioned low SNR, partially due to the choice of using a mouse model on a human clinical MRI scanner. While techniques such as compressed sensing^40^ can be combined with ^19^F MRI to improve its sensitivity, such a limitation should be overcome once applied to humans with the assumption of increased PFC uptake and plaque volume: given that much larger voxel volumes can be used, a significantly higher ^19^F SNR than that measured in this study can be expected. Due to an already lengthy acquisition protocol, magnetic field (B_0_) and RF transmission (B_1_) field maps were not acquired *in vivo*, although use of the birdcage RF coil should limit B_1_ inhomogeneities. These limitations indicate that several further critical translational steps (including regulatory approval) will need to be made in future studies to make ^19^F MRI of atherosclerotic inflammation a clinical reality.

The exact mechanism by which the PFC is taken up by the immune system and is transported to the inflammation site could not be determined in this study. To gain insight into such mechanisms, future studies might include PFC nanoparticles that are adapted for intravital multiphoton microscopy^41,42^, which can be used to track the fate of individual PFC-loaded leukocytes.

In conclusion, we demonstrated that ^19^F MRI allows for the detection and quantification of the PFC in atherosclerotic plaque *in vivo* on a 3 T clinical scanner. The PFC was taken up primarily by dendritic cells and only in small amount by macrophages or neutrophils. Considering the impact that inflammation has on atherosclerosis development and progression, this translational step will help set up ^19^F MRI of PFCs as a tool to longitudinally quantify inflammation and thus to guide therapies and to monitor the inflammation.

## Methods

The data that support the findings of this study are available from the corresponding author upon reasonable request.

### Animal model

**Figure 6:**
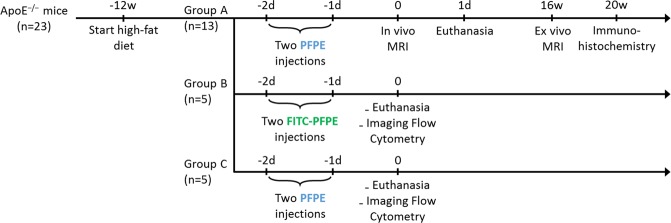
Experimental design and animal groups. All animals were fed a high-fat diet for 12 weeks. All mice received two intravenous PFC injections, two and one days before MRI or imaging flow cytometry experiments. Mice from group A and C received PFPE while mice from group B received FITC-PFPE. All groups were sacrificed at day one. Animals from group A were scanned *ex vivo* afterward.

All animal studies were approved by the local animal ethics committee (Veterinary Affairs of the Canton Vaud authorization VD2957). All experiments were carried out in accordance with the relevant guidelines and regulations. Five-week-old male C57BL/6 apolipoprotein E-knockout (ApoE^−/−^) mice (n = 23, Charles River Laboratories, L’Arbresle, France), which are prone to develop atherosclerosis^43^, were fed a high-fat diet (including 20% milk butter and 0.15% cholesterol, Safe Diets, Augy, France) for 12 weeks to exacerbate atherosclerotic plaque development (Figure 6).

For the MR studies, thirteen ApoE^−/−^ mice (group A) received 2 × 200 µL PFPE (V-Sense VS-1000H, Celsense Inc, Pittsburgh, Pennsylvania, USA) through the tail vein two and one days before imaging (Figure 6). This PFC is characterized by a single narrow ^19^F resonance, while the average nanoparticle size is between 160 and 190 nm^44^. Since previous studies^16^ demonstrated the complete absence of ^19^F signal in control mice at the level of the aortic arch and its branches, i.e. the brachiocephalic, left subclavian and left carotid arteries, no MRI control group was included in the experimental design. For the imaging flow cytometry^23^, group B and C (5 mice each) received the same 2 × 200 µL injection protocol with fluorescein isothiocyanate PFPE (FITC-PFPE, V-Sense VS-1000H DM Green, Celsense Inc, Pittsburgh, Pennsylvania, USA) and regular PFPE (as control), respectively.

### *In vivo* MR imaging

All MR experiments were performed on a 3 T clinical system (MAGNETOM Prisma, Siemens Healthineers, Erlangen, Germany). A 35 mm-diameter transmit/receive birdcage radiofrequency (RF) coil tunable to both the ^19^F and ^1^H frequencies (Rapid Biomedical, Rimpar, Germany) was used for both excitation and signal detection.

Two days after the first injection, the animals were anesthetized with 2.5% isoflurane in oxygen for 1–2 minutes. Anesthesia was maintained with 1.5–2% of isoflurane for the duration of the scan. The heart rate was monitored through an electrocardiogram (ECG) obtained with electrodes placed on the paws, the body temperature was monitored with a rectal probe and maintained at 37 ± 0.5 °C with a tubing system with circulating hot water, while the respiratory activity was monitored by means of a respiration pillow placed below the mouse abdomen (all SA Instruments, Stony Brook, NY, USA). An external reference tube with a known concentration of PFPE (22 mM –resulting in a ^19^F concentration of 1.05 M) in 2% weight/volume agar gel was created and used for absolute quantification. The tube was carefully placed diagonally above and besides the thorax of the animal such that its artifacts along the phase encoding directions could not occur inside the thorax.

3D gradient-recalled echo (GRE) ^1^H images were acquired for anatomic localization with the following MR imaging parameters: repetition time TR = 7.6 ms, echo time TE = 3.3 ms, pixel bandwidth BW = 395 Hz/pixel, RF excitation angle α = 20°, field of view FOV = 100 × 100 × 32 mm^3^, voxel size = 0.3 × 0.3 × 0.5 mm^3^, number of slices = 64, number of averages NA = 5, acquisition time T_acq_ = 9min35s. In n = 8 mice a respiratory-gated and ECG-triggered segmented 2D bright-blood ^1^H GRE pulse sequence was acquired for visualization of the aorta and aortic arch branches with the following parameters: TR = 129 ms, TE = 2.84 ms, BW = 415 Hz/pixel, α = 60°, FOV = 120 × 82 mm^2^, pixel size = 0.27 × 0.27 mm^2^, slice thickness = 1 mm, number of slices = 3, NA = 4, T_acq_ = ~3 min per slice.

^19^F MR images were obtained with a 3D turbo spin echo (TSE) pulse sequence with the following sequence parameters optimized for maximal SNR in 30 minutes^38^: TR = 1070 ms, TE = 13 ms, BW = 130 Hz/pixel, ETL = 13, FOV = 100 × 100 × 32 mm^3^, voxel size = 0.8 × 0.8 × 1 mm^3^, number of slices = 32, NA = 6, T_acq_ = 28 min 52 s; partial Fourier factor in slice-encoding direction = 3/4. The center of the 3D volume was placed at the same position as the one used for the ^1^H 3D GRE pulse sequence.

### *Ex vivo* photography and imaging

The day after the *in vivo* scans, all mice from group A were euthanized (Figure 6). The hearts and aortas were then perfused with cold phosphate-buffered saline (PBS) through the left ventricle to flush out the blood. The aortas, including the aortic arch branches, were excised and placed in a petri dish containing cold PBS, where excess connective tissue and fat was removed under a stereo microscope. Finally, the aortas were fixed in 10% neutralized formalin. In order to visually locate the atherosclerotic plaques, en face photographs of each aorta were taken with a digital camera (Coolpix, Nikon, Tokyo, Japon), and the average plaque surface was measured on the resulting photos.

*Ex-vivo* 3D GRE ^1^H images were acquired of the excised aortas with the following parameters: TR = 40 ms, TE = 4.17 ms, BW = 255 Hz/pixel, α = 25°, FOV = 100 × 100 × 32 mm^3^, voxel size = 0.4 × 0.4 × 0.5 mm^3^, number of slices = 64, NA = 6, T_acq_ = 1 h 5 min 32 s.

^19^F images were acquired with the ^19^F pulse sequence used for *in vivo* scans, with NA = 110, T_acq_ = 11 h 46 min 11 s, and without partial Fourier factor in slice-encoding direction. As was done for *in vivo* scans, the center of the 3D volume was placed at the same position as that of the ^1^H pulse sequence to facilitate ^1^H/^19^F image coregistration and overlay. *Ex vivo* ^19^F images were used for confirmation of the presence of ^19^F signal in the aortas only: no SNR measurements were performed due to the long time period (~16 weeks) between the fixation of the aortas and the available scanning sessions.

### MR image reconstruction and processing

MR image reconstruction and analysis were performed in Matlab (MathWorks, Natick, Massachusetts, USA). The raw ^19^F MR data of all thirteen *in-vivo* mouse acquisitions were reconstructed with both a standard Fourier transformation and a denoised reconstruction. The denoised reconstruction was performed with an iterative algorithm that behaved as a wavelet denoising filter^45^:

1 $${\rm{\arg }}\,{\min }_{m}{\Vert Fm-y\Vert }_{2}^{2}+{\lambda }_{\Psi }{\Vert \Psi m\Vert }_{1},$$

where *F* is the Fourier operator, *m* is the reconstructed image, *y* is the acquired raw data, Ψ is the wavelet operator (Debauchies-2 wavelet), and λ_Ψ_ is the matching regularization parameter, which was set to 0.008. The number of iterations was empirically established at 32 to ensure sufficient convergence of the algorithm.

In the images reconstructed with the standard Fourier transformation, the threshold to separate ^19^F signal from noise was fixed as a multiple of the standard deviation of the background noise measured well apart from the mouse or the external reference, and such that no ^19^F signal was left outside the mouse body. The same threshold was applied to the denoised images. Given that ^1^H and ^19^F acquisitions did not have the same spatial resolution, 3D ^19^F images were linearly interpolated to overlay the ^1^H images for visualization purposes. For both the *in vivo* and *ex vivo* scans, the external reference tube was used as a control for ^1^H/^19^F image alignment. ^1^H *ex vivo* images underwent a multiplanar reformat with Soap-Bubble^46^ in order to project the aorta onto a single plane.

The quantification of the ^19^F signal in suspected atherosclerotic plaques was performed on the non-interpolated images. The SNR of an ROI was defined as the ratio between the average signal intensity (SI) in the ROI and the standard deviation of a region of noise well outside the body and the external reference tube. In images that were reconstructed without the denoising filter, the ^19^F SNR, the volume of the segmented ^19^F signal and their product, i.e. the ^19^F signal integral, were calculated. The MRI-derived PFC concentration was calculated as the ratio of the SNR of the ROI and that of the external reference. In images that were reconstructed with the denoising filter, the volume of the segmented ^19^F signal and the MRI-derived PFC concentration were calculated. Given that the background noise of images that have been denoised depends highly on the regularization parameters chosen for the reconstruction, no meaningful SNR calculation could be performed. Therefore, the MRI-derived PFC concentration was calculated by referencing the SI of the ROI to that from the external reference. Given that a birdcage RF coil and low regularization weights were used, the two were assumed to scale linearly. In the absence of a control group, the SNR was also measured in the aortic arch in regions without a clear ^19^F MR signal in each of the thirteen standard-reconstructed 3D images.

Fiji^47^ was used to manually segment the atherosclerotic plaques from the anterior and posterior en face photographs of each aorta, and the visible surface of the plaques was estimated with the help of small tape measure.

### Imaging flow cytometry

After 12 weeks of high-fat diet, all animals from group B and C were euthanized. Aortas were gently perfused with cold PBS through the left ventricle to remove the blood, carefully exercised, and cleaned for excess connective tissue and fat. The specimens were then dissected in small pieces and digested for 40 minutes at 37 °C in 1% fetal calf serum (FCS, Sigma-Aldrich, Saint Louis, Missouri, USA) dissociation enzyme solution. Following the incubation, single-cell suspensions were obtained by means of filtering through a 70 µm nylon mesh into 5 ml polypropylene tubes. The cells were then spun down at 350 g for 10 minutes to form a pellet. Next, trypan blue staining and light microscopy were used for cell counting, and finally the samples were incubated on ice with the appropriate combination of antibodies. The used antibodies included: anti-F4/80, anti-CD45, anti-Ly6C, anti-CD11c, anti-CD11b and anti-Gr1 (all from Biolegend, San Diego, California, USA). The Live/Dead Fixable Aqua Dead Cell Stain kit (Aqua) and 4’,6-diamidino-2-phenylindole (DAPI, ThermoFisher Scientific, Waltham, Massachusetts, USA), were used to mark dead cells and cell nuclei, respectively.

Individual cell images were acquired using INSPIRE software (Amnis Merck Millipore, Billerica, Massachusetts, USA) on a 4-laser 12-channel imaging flow cytometer (Image Stream X Mark II, Amnis Merck Millipore) with 40× magnification. Prior to each experiment, the machine was fully calibrated using the ASSIST user interface (Amnis Merck Millipore). For each data file, at least 50000 single cells were acquired - debris and doublets were excluded based on their area and aspect ratio. Single-stain controls were acquired (all channels on, no brightfield and no side scatter image), a compensation matrix was calculated and then applied to the data files using IDEAS software (Amnis Merck Millipore).

To analyze the data files, the cells in focus (using the root mean square of the rate of change of the image intensity profile for the bright-field image) and the single cells (using the area-aspect ratio plot) based on the bright-field and DAPI images were gated. Within the Aqua negative cells (live cells), FITC-PFPE positive cells (using the intensity-maximum pixel FITC-PFPE plot) were selected. The expression of the different surface markers was used to identify the various cell populations.

The gating strategy was as follows^48–50^: CD45 positive (+) and negative (−) cells were gated. Within the CD45+ population, three distinct populations were found by staining Gr1 versus CD11b. The Gr1+ CD11b+ population was then examined for Ly6C expression, and Ly6C+ cells were then defined as neutrophils. CD11b+ Gr1− and CD11b− Gr1− cells were examined for the dendritic cell markers CD11c and F4/80. The CD11b+ Gr1− CD11c− F4/80− population was defined as macrophages, while the CD11b− Gr1− CD11c+ population was defined as dendritic cells. The CD45+ CD11b− Gr1− CD11c− F4/80− Ly6C− population was considered as lymphocytes. We measured whether the PFC signal was localized inside or outside the cells using the internalization feature of FITC-PFPE in an erode mask of the bright-field image plotted against the delta centroid (a compound feature that is used to measure the separation between two fluorescent probes) between the FITC-PFPE and bright-field images. By means of the Aqua expression, we eliminated the dead cells.

For each cell, a mask on the largest FITC-PFPE spot was automatically drawn and the area was calculated in µm^2^. The relative brightness of this spot was calculated as the sum of the individual pixel intensity values, and the area-brightness product was defined as the total PFC signal per cell. The total number of FITC-PFPE+ cells was also reported.

### Immunohistochemistry

Based on the results of the IFC analysis, immunohistochemistry was performed to identify leukocytes, macrophages and dendritic cells in plaques. The brachiocephalic artery, a site of predilection for the development and progression of atherosclerotic plaques in high-fat-diet-fed ApoE^−/−^ mice^51,52^, of the thirteen formalin-fixed aortas (group A) was embedded in paraffin and cross-sectioned at the level of the plaque in 3 µm thick slices. Slices were then immunostained for macrophages with a monoclonal Mac-2 antibody (Cedarlane Labs, Ontario, Canada, dilution 1/200), for leukocytes with a polyclonal CD45 antibody (Abcam, Cambridge, UK, dilution 1/100) and for dendritic cells with a polyclonal S100 antibody (Agilent Dako, Santa Clara, California, USA, dilution 1/1000). Subsequently, the sections were incubated with the appropriate secondary antibody. For the Mac-2 staining, antibodies were revealed with a peroxidase-linked avidin-biotin detection system as previously described^53^. For the CD45 and S100 stainings, antibodies were revealed with the peroxidase-conjugated EnVision + reagent (HRP Rabbit, K4003, Agilent Dako). For each immunostaining, three slices were selected at three different levels of the plaque in order to obtain an overview of the inflammatory state: at the center of the plaque, at the beginning, and at the end. In total, nine slices (3 stains × 3 locations) were stained per mouse. Several extra slices were prepared with appropriate isotype controls to ensure for the specificity of each immunostaining. Images were acquired under a Leica DMLB microscope connected to a Leica DC300F camera (Leica Microsystems, Wetzlar, Germany), and quantification was performed under light microscopy with Leica QWin software (Leica Microsystems, Wetzlar, Germany)^53^. The total plaque area was calculated for each stained slice, and the expression of antigens was quantified by measuring positive stained areas or cell counting. Results were expressed either as positive-stained area over total plaque area (Mac-2 and CD45) or total number of positive-cells per plaque area (S100)^53,54^. Since three slices were analyzed per plaque, each immunohistochemistry parameter was calculated for the average value of the three slices and reported averaged over the thirteen brachiocephalic artery plaques.

### Statistical analysis

A Shapiro-Wilk normality test was used to assess the normal distribution of all continuous variables. Variables assessed as normally distributed are reported as mean ± one standard deviation. Linear correlations were assessed by calculating the Pearson’s correlation coefficient r and the corresponding p-value. Variables assessed as non-normally distributed are reported as median (IQR = quartile 1 − quartile 3). Linear correlations were performed by calculating the Spearman’s rank correlation coefficient ρ and the corresponding p-value. Linear correlations were investigated between ^19^F MRI metrics, en face photo analyses and immunohistochemistry metrics. For *in vivo* quantification and immunohistochemistry analysis, for normally distributed data a paired Student’s t-test was used to account for significant differences, while a Wilcoxon matched-pairs signed rank test was performed on the non-normally distributed data. For all tests, P < 0.05 was considered significant, although for the correlations only so if their correlation coefficient was positive. A Bonferroni correction for multiple comparisons was applied when required.

## Data Availability

The datasets that were generated and analyzed during the current study are available from the corresponding author on reasonable request.
